# Prefrontal gamma oscillations reflect ongoing pain intensity in chronic back pain patients

**DOI:** 10.1002/hbm.24373

**Published:** 2018-09-10

**Authors:** Elisabeth S. May, Moritz M. Nickel, Son Ta Dinh, Laura Tiemann, Henrik Heitmann, Isabel Voth, Thomas R. Tölle, Joachim Gross, Markus Ploner

**Affiliations:** ^1^ Department of Neurology Technische Universität München Munich Germany; ^2^ TUM‐Neuroimaging Center Technische Universität München Munich Germany; ^3^ Institute of Neuroscience and Psychology University of Glasgow Glasgow United Kingdom; ^4^ Institute for Biomagnetism and Biosignalanalysis University of Münster Münster Germany

**Keywords:** brain, chronic pain, electroencephalography, oscillations, prefrontal cortex

## Abstract

Chronic pain is a major health care issue characterized by ongoing pain and a variety of sensory, cognitive, and affective abnormalities. The neural basis of chronic pain is still not completely understood. Previous work has implicated prefrontal brain areas in chronic pain. Furthermore, prefrontal neuronal oscillations at gamma frequencies (60–90 Hz) have been shown to reflect the perceived intensity of longer lasting experimental pain in healthy human participants. In contrast, noxious stimulus intensity has been related to alpha (8–13 Hz) and beta (14–29 Hz) oscillations in sensorimotor areas. However, it is not fully understood how the intensity of ongoing pain as the key symptom of chronic pain is represented in the human brain. Here, we asked 31 chronic back pain patients to continuously rate their ongoing pain while simultaneously recording electroencephalography (EEG). Time–frequency analyses revealed a positive association between ongoing pain intensity and prefrontal beta and gamma oscillations. No association was found between pain and alpha or beta oscillations in sensorimotor areas. These findings indicate that ongoing pain as the key symptom of chronic pain is reflected by neuronal oscillations implicated in the subjective perception of longer lasting pain rather than by neuronal oscillations related to the processing of objective nociceptive input. The findings, thus, support a dissociation of pain intensity from nociceptive processing in chronic back pain patients. Furthermore, although possible confounds by muscle activity have to be taken into account, they might be useful for defining a neurophysiological marker of ongoing pain in the human brain.

## 1 INTRODUCTION

Chronic pain is a pathological condition characterized by ongoing pain and a range of sensory, cognitive, and affective abnormalities (Moriarty, McGuire, & Finn, 2011; Velly & Mohit, 2018). It affects between 20% and 30% of the population and represents a large burden to patients and health care systems (Rice, Smith, & Blyth, 2016). Its treatment is often difficult (Maher, Underwood, & Buchbinder, 2017), partially due to an incomplete understanding of underlying neural mechanisms.

The brain plays a central role in chronic pain. Many studies have assessed the persisting characteristics of the pathological chronic pain state by comparing brain structure and brain function between chronic pain patients and healthy participants. They revealed that chronic pain is associated with extensive changes of brain structure and function (Baliki & Apkarian, 2015; Kuner & Flor, 2017; Pinheiro et al., 2016; Rauschecker, May, Maudoux, & Ploner, 2015), which consistently involve prefrontal and limbic structures.

Fewer studies have explicitly investigated how the intensity of ongoing pain as the key symptom of chronic pain is represented in the human brain. Such brain markers of ongoing pain intensity are of particular interest as they constitute potential targets for pain treatment using approaches such as neurofeedback and neurostimulation (Jensen, Day, & Miro, 2014; Sitaram et al., 2017; Thut et al., 2017). Functional magnetic resonance imaging (fMRI) studies of different chronic pain populations have shown that ongoing pain intensity is reflected by blood‐oxygen level dependent (BOLD) signals in the medial prefrontal cortex (Baliki et al., 2006; Geha et al., 2007; Parks et al., 2011). However, the BOLD effect is an indirect measure of neuronal activity, which does not differentiate between neuronal activity at different frequencies. Neuronal oscillations at different frequencies represent fundamental features of neuronal signaling and communication (Buzsaki & Draguhn, 2004; Donner & Siegel, 2011; Fries, 2015; Wang, 2010) and can be individually targeted using neuromodulation methods including neurofeedback (Jensen et al., 2014; Sitaram et al., 2017; Thut et al., 2017). The direct neuronal correlate and the frequency profile of the encoding of ongoing pain in chronic pain is still unknown.

Electroencephalography (EEG) directly measures neuronal activity at different frequencies. As a first approximation of ongoing pain in chronic pain, we recently investigated the neurophysiological encoding of ongoing experimental pain in healthy human participants using EEG (Nickel et al., 2017; Schulz et al., 2015). These studies revealed a detachment of perceived pain intensity from noxious stimulus intensity already within a few minutes. Moreover, they showed that objective noxious stimulus intensity was inversely related to alpha (8–13 Hz) and beta (14–29 Hz) oscillations in sensorimotor areas, whereas subjective pain was positively related to neuronal oscillations at gamma (60–90 Hz) frequencies in the prefrontal cortex.

Here, we hypothesized that the intensity of ongoing pain in chronic pain is reflected by neuronal activity related to the perception of longer lasting pain, that is, prefrontal gamma oscillations, rather than neuronal activity related to nociceptive processing, that is, alpha and beta oscillations in sensorimotor areas. We asked chronic back pain patients to continuously rate their ongoing pain while recording EEG. Time–frequency analyses revealed that ongoing pain intensity is reflected by prefrontal gamma oscillations but not by alpha and beta oscillations in sensorimotor areas. These findings hint at a direct neurophysiological marker of ongoing pain as the key symptom of chronic pain. Furthermore, they provide physiological support for a dissociation of ongoing pain from nociceptive processes in chronic pain.

## 2 MATERIALS AND METHODS

### 2.1 Participants

Thirty‐one chronic back pain patients were included in the final sample of the study (age 56 ±13 years [mean ± standard deviation], 17 females, 30 right‐handed). Data from five additional participants were not further analyzed as they did not report pain during the recording. General inclusion criteria were a clinical diagnosis of chronic pain with the focus of pain in the back, a duration of pain ≥6 months and a minimum reported average pain intensity ≥4/10 during the last 4 weeks (0 = *no pain*, 10 = *worst imaginable pain*). Participants were excluded if there had been acute changes of the pain condition during the last 3 months, for example, due to recent injuries or surgeries. Further exclusion criteria were major neurological diseases such as strokes, epilepsy, or dementia, major psychiatric conditions aside from depression, and severe internal diseases. Finally, patients on medication with benzodiazepines were excluded. For pain treatment, 14 patients took antidepressants (5 selective serotonin/noradrenaline reuptake inhibitors, 8 tri/tetracyclic antidepressants, and 1 other), 14 GABAergic anticonvulsants, 14 nonsteroidal anti‐inflammatories, and 10 opioids. In addition to a clinical examination, patients were characterized using a range of clinical questionnaires including the Medication Quantification Scale (MQS) (Harden et al., 2005), the Beck Depression Inventory II (BDI) (Beck, Steer, & Brown, 1996), the State‐Trait Anxiety Inventory (STAI) (Spielberger, Gorsuch, Lushene, Vagg, & Jacobs, 1983), the short‐form McGill pain Questionnaire (SF‐MPQ) (Melzack, 1987), the Roland Morris Disability Questionnaire (RMDQ) (Roland & Morris, 1983), and the painDETECT questionnaire (Freynhagen, Baron, Gockel, & Tolle, 2006). Please see Table 1 for detailed patient characteristics. The nature of the experimental procedures was explained to all participants and all gave written informed consent. The study was approved by the ethics committee of the Medical Faculty of the Technische Universität München and carried out in accordance with the relevant guidelines and regulations.

**Table 1:** Patient characteristics

Subject	Age (years)	Gender	Pain duration (yeas)	Current pain intensity (VAS, 0–100)	Pain location	Pain origin	MQS	BDI	SF‐MPQ sensory	SF‐MPQ affective	STAI state	STAI trait	RMDQ	painDETECT
1	67	m	30	49	Lumbar	Radiculopathy	4	3	13	0	27	27	5	28
2	54	f	10	36	Lumbar	Radiculopathy	32	35	8	2	56	54	16	13
3	64	f	8	65	Lumbar	Radiculopathy	11	7	11	2	47	37	13	13
4	41	m	2	69	Lumbar, cervical	Radiculopathy, vertebral fracture (traumatic)	6	15	16	2	33	36	9	21
5	74	m	15	34	Lumbar	Myelopathy (spinal stenosis)	7	18	5	2	35	35	12	5
6	58	f	14	69	Lumbar	Radiculopathy	19	22	20	7	67	48	17	18
7	65	m	4	64	Lumbar	Vertebral fracture (osteoporotic)	4	11	4	0	55	37	6	10
8	65	f	11	24	Lumbar	Radiculopathy	6	5	8	0	32	31	10	23
9	76	f	2	56	Lumbar	Radiculopathy	4	9	12	2	31	36	12	19
10	33	f	3	60	Lumbar	Radiculopathy	9	19	17	3	36	48	16	14
11	45	f	1	71	Lumbar, cervical	Musculoskeletal	5	20	14	4	50	56	9	6
12	51	f	2	47	Lumbar	Radiculopathy	11	22	7	3	61	56	15	16
13	73	f	9	70	Lumbar	Radiculopathy	11	21	15	5	44	51	7	25
4	41	f	10	50	Lumbar	Musculoskeletal	13	14	13	3	47	50	8	12
15	55	f	21	89	Lumbar	Radiculopathy	24	24	17	9	54	50	18	18
16	73	m	25	45	Lumbar	Vertebral fracture (traumatic)	4	10	9	2	35	35	15	6
17	46	m	30	63	Thoracic	Radiculopathy, scoliosis	16	10	10	3	39	41	9	4
18	50	m	5	67	Cervical	Myelopathy (disc herniation)	0	5	13	4	21	37	4	18
19	59	f	2	53	Lumbar	Radiculopathy	12	31	21	10	33	60	9	22
20	62	m	1	54	Lumbar	Degenerative (spondylarthrosis)	10	26	6	4	59	60	15	21
21	54	m	7	54	Lumbar, cervical	Myelopathy (spinal stenosis)	16	18	10	2	30	47	13	16
22	39	f	12	40	Cervical	Musculoskeletal	0	12	21	4	44	43	4	19
23	66	m	4	45	Lumbar	Degenerative (spondylarthrosis)	8	19	7	3	48	46	14	9
24	57	m	25	49	Lumbar	Radiculopathy	22	12	17	8	52	51	17	16
25	52	m	25	23	Lumbar	Degenerative (spondylolisthesis)	11	22	13	5	35	57	9	11
26	47	f	15	32	Thoracic	Degenerative (spondylarthrosis)	26	19	16	3	49	59	8	12
27	24	f	8	75	Lumbar	Degenerative (spondylolisthesis)	5	13	13	6	42	46	12	20
28	59	f	15	30	Lumbar	Radiculopathy	6	4	13	4	38	43	10	9
29	82	m	5	50	Lumbar	Degenerative (spondylolisthesis), myelopathy (spinal stenosis)	14	21	6	2	51	49	16	7
30	54	m	10	54	Lumbar	Radiculopathy	12	17	20	9	44	46	13	20
31	62	w	3	35	Lumbar	Degenerative (spondylarthrosis)	3	22	7	2	35	48	11	10
Mean	56.39		10.70	52.33			10.64	16.32	12.32	3.71	42.90	45.81	11.35	14.87
Sd	13.16		8.86	15.94			7.47	7.79	4.91	2.61	10.96	8.87	4.00	6.26

*Note.* Abbreviations: BDI = Beck Depression Inventory II; f = female; m = male; MQS = Medication Quantification Scale; SF‐MPQ = short‐form McGill Pain Questionnaire; RMDQ = Roland Morris Disability Questionnaire; STAI = State–Trait Anxiety Inventory; sd = standard deviation; VAS = visual analogue scale; ys = years.

Current pain intensity was assessed by questionnaires completed immediately before the EEG experiment.

### 2.2 Experimental design

The experiment consisted of two conditions; a *spontaneous pain* and a *visual control* condition, which were recorded consecutively with a short break in between. During both conditions, participants were comfortably seated in front of a computer screen and wore headphones playing white noise to mask ambient noise. Both arms were comfortably placed on arm rests. During the *spontaneous pain* condition, participants were asked to attentively monitor their ongoing pain for 11 min and continuously rate the current pain intensity on a visual analogue scale (VAS) anchored at *no pain* and *worst imaginable pain* using a custom‐built finger‐span device with the right hand. The scale was simultaneously presented on a screen by a vertical red bar, the length of which represented the current pain intensity rating. Pain was primarily localized in the back sometimes extending to other body parts. Patients were asked to provide an overall rating of pain intensity regardless of its current location. The *visual control* condition was performed to control for activity related to the continuous rating procedure such as visual‐motor performance, magnitude estimation, and anticipation (Baliki, Baria, & Apkarian, 2011; Hashmi et al., 2013; Nickel et al., 2017). Unbeknownst to the subject, in this condition, 10 min of the time course of the individual pain rating from the *spontaneous pain* condition were visually presented on the screen as changes of the length of the vertical red bar over time. Participants were instructed to continuously track the length of the bar, again using the custom‐built finger span device with their right hand. The first minute of the spontaneous pain rating time course was omitted to leave out the initial positioning of the red bar to the current pain intensity. As this condition used the pain rating time course from the *spontaneous pain* condition, the *spontaneous pain* condition was always performed first.

To become familiar with the procedures, all patients performed 5 min practice runs of each condition, using a predefined time course of bar length changes for the practice run of the *visual control* condition. Stimulus presentation and timing was controlled using Matlab (Mathworks, Natick, MA) and the Psychophysics Toolbox (http://psychtoolbox.org/).

### 2.3 Recordings

EEG data were recorded using an electrode montage of 64 electrodes consisting of all 10–20 system electrodes and the additional electrodes Fpz, CPz, POz, Oz, Iz, AF3/4, F5/6, FC1/2/3/4/5/6, FT7/8/9/10, C1/2/5/6, CP1/2/3/4/5/6, TP7/8/9/10, P5/6, and PO1/2/9/10 plus 2 electrodes below the outer canthus of each eye (Easycap, Herrsching, Germany) and BrainAmp MR plus amplifiers (Brain Products, Munich, Germany). All EEG electrodes were referenced to FCz and grounded at AFz. Simultaneously, muscle activity was recorded with 2 bipolar surface electromyography (EMG) electrode montages placed on the right masseter and neck (semispinalis capitis and splenius capitis) muscles (Davis, 1959) and a BrainAmp ExG MR amplifier (Brain Products, Munich, Germany). EMG electrodes were grounded at the cervical vertebra C2. All data were sampled at 1,000 Hz (0.1 μV resolution) and band‐pass filtered between 0.016 Hz and 250 Hz. Impedances were kept below 20 kΩ. In addition, continuous (pain) ratings were fed into the EEG system and recorded with the same sampling frequency.

### 2.4 Preprocessing

Preprocessing was performed using the BrainVision Analyzer software (Brain Products, Munich, Germany). Data were downsampled to 500 Hz. For artifact identification, a high‐pass filter of 1 Hz and a 50 Hz notch filter for line noise removal were applied to the EEG data. Independent component analysis was then applied (Jung et al., 2000) and components representing eye movements and muscle artifacts were identified based on component time courses and their topographical distribution. Furthermore, time intervals of 400 ms around data points with amplitudes exceeding ±100 μV and signal jumps exceeding ±30 μV were marked for rejection. Last, all data were visually inspected and additional bad segments marked. Subsequently, independent components representing artifacts were subtracted from the raw, unfiltered EEG data (Winkler, Debener, Muller, & Tangermann, 2015) and EEG data were re‐referenced to the average reference. In all analyses, data from min 3 to min 11 from the spontaneous pain and from min 2 to min 10 from the *visual control* condition were used, resulting in a total of 9 min per condition. Thus, corresponding sections of both conditions were selected while excluding initial adjustments of the rating at the beginning of each condition. EMG electrodes were not included in the artifact rejection procedure, but intervals previously marked as bad based on the EEG data were omitted from all further analyses of both EEG and EMG data. A control analysis of the *spontaneous pain* condition did not show a significant relationship between pain ratings and the percentage of data rejected (mean *r* = −.06, Pearson correlation; *p* = .55, *t* test vs. 0).

### 2.5 Relationships between chronic pain intensity and brain activity

All further analyses were performed using the FieldTrip toolbox (Oostenveld, Fries, Maris, & Schoffelen, 2011), custom programming in Matlab, and IBM SPSS Statistics for Windows (SPSS), version 25 (IBM Corp., Armonk, NY). The main goal of our analyses was to relate spontaneous fluctuations of the ongoing pain intensity to neuronal activity in different frequency bands.

#### 2.5.1 Electrode space analysis

For each subject, EEG data of the *spontaneous pain* condition were first bandpass‐filtered in theta (4–7 Hz), alpha (8–13 Hz), beta (14–29 Hz), and gamma (60–90 Hz) frequency bands using a fourth‐order Butterworth filter (forward and backward). To obtain time courses of amplitude changes in the different frequency bands, that is, amplitude envelopes, absolute values of the Hilbert transform were computed. These envelopes and the raw pain ratings were then further downsampled and smoothed using a moving average with a window length of 1 s and a step size of 0.1 s. For each electrode, the amplitude envelopes were subsequently z‐transformed across the whole time series and sorted according to the rating of the current pain intensity at each data point. Then, five equally large bins of data were formed comprising the 20% of data with the lowest pain ratings (bin 1) up to the 20% of data with the highest pain ratings (bin 5). For each subject, relationships between EEG data and the currently perceived pain intensity were then quantified per electrode and frequency band using linear regressions based on the bin label (1–5) and the averaged z‐transformed amplitude in each bin. Thus, regressions were based on five data points per electrode and frequency band. For display purposes and statistics (see below), relationships were quantified across participants as dependent‐samples regression *t* statistics by dividing the mean regression coefficients by their standard errors (Litvak et al., 2007; Lorch & Myers, 1990). To investigate the effect of equalizing pain rating variations across subjects, the analysis was also performed without z‐transformation.

**Figure 1:**
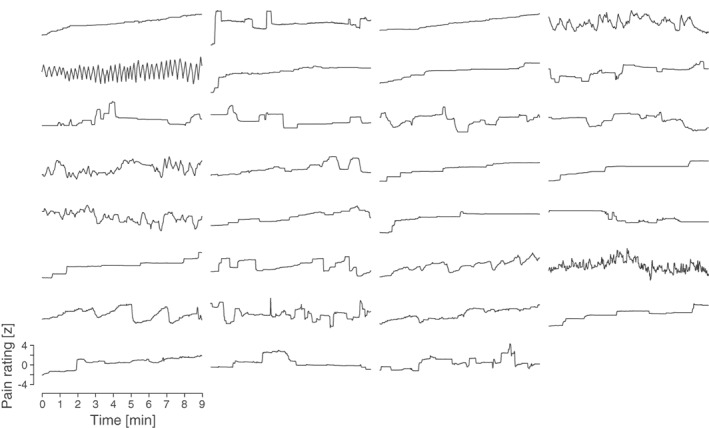
Single‐subject spontaneous pain ratings. Individual pain ratings are shown for the analyzed time window. Pain intensity was continuously rated on a visual analogue scale anchored at *no pain* and *worst imaginable pain.* Ratings were smoothed using a moving average with a window length of 1 s and a step size of 0.1 s. Ratings were subsequently *z*‐transformed to account for varying strength of pain rating fluctuations across participants.

Since many patients showed a slow increase of pain in the *spontaneous pain* condition over the course of the experiment (Figure 1) and mean pain ratings significantly increased over time (see below), we performed two further analyses investigating the contribution of time to our observed results. First, we repeated the electrode space analysis separately for the first and last 4.5 min of the analyzed time window. Second, we included time as a covariate in our analysis. The latency since the beginning of the recording was averaged for each of the five bins and included as additional predictor in all regressions.

#### 2.5.2 Frequency resolved analysis

To show the frequency spectrum of the relationship between brain activity and perceived pain intensity in the *spontaneous pain* condition, an additional analysis was performed. This analysis focused on the fronto‐central electrode Fz, which was part of the significant cluster in the electrode space analysis. Matching the moving average approach from the previous analysis, preprocessed EEG data at electrode Fz were segmented into 1 s segments with 90% overlap. After applying a Hanning taper, power was estimated for frequencies between 1 and 100 Hz in steps of 1 Hz for each segment using the Fast Fourier Transform (FFT). Subsequently, the power was z‐transformed across all segments for each frequency. Average pain ratings of all 1 s segments were used to sort the power spectra into five equally large bins. These power spectra were then again averaged over the segments, resulting in a single power spectrum for each bin. Last, to quantify the frequency resolved relationships between brain activity and pain ratings across participants, dependent‐samples regression *t*‐values were computed again, now calculating linear regression coefficients between bin labels and power averages for each 1‐Hz‐frequency step and then dividing their means by the standard errors across participants. Please note that this analysis was only performed for visualizing the frequency spectrum of the relationships between ongoing pain and neuronal oscillations without repeating statistical group analysis.

#### 2.5.3 Trend analysis

To confirm that our linear regression approach captured the prevailing type of relationship between ongoing pain and neuronal oscillations, we additionally performed a trend analysis at electrode Fz for the *spontaneous pain* condition. This analysis was performed for the gamma frequency band only, which was the only frequency band showing a significant relationship with ongoing pain in the previous electrode space analysis. Again, we averaged *z*‐transformed gamma activity in the five bins based on pain ratings and then performed a repeated measures analysis of variance (ANOVA) with subsequent trend analysis.

#### 2.5.4 Source level analysis

In the next step, relationships between ongoing pain intensity and brain activity were quantified on source level. Source analysis maps EEG signals to the brain and has been shown to reduce muscle and ocular artifacts (Hipp & Siegel, 2013; Muthukumaraswamy, 2013). In line with the electrode level analysis, source analysis was performed for theta, alpha, beta, and gamma frequency bands. Using linearly constrained minimum variance (LCMV) beamforming (Van Veen, van Drongelen, Yuchtman, & Suzuki, 1997), band‐pass filtered data in each frequency band were projected from electrode to source space for each subject. Individual spatial filters were computed based on the average covariance matrices across nonoverlapping 1 s segments of the preprocessed and band‐pass filtered data of all pain rating bins and a regularly spaced 3D grid with a 1 cm resolution. The leadfield was computed for each voxel using a realistically shaped three‐shell boundary‐element volume conduction model based on the template Montreal Neurological Institute (MNI) brain. A regularization parameter of 5% was used and the dominant dipole orientation was chosen. By projecting EEG data through the spatial filter, time courses of neuronal activity per frequency band were obtained for each voxel, which were then analyzed in parallel to the electrode level analysis. For each voxel, the amplitude envelope was computed using the Hilbert transform. This envelope was then downsampled using the moving average and z‐transformed across the whole time series. Average amplitudes within five equally large data bins based on the sorted pain ratings was calculated for each voxel and linear regression coefficients between average amplitudes and bin labels were computed per subject and then summarized across participants using dependent‐samples regression *t* statistics.

### 2.6 Control analyses

#### 2.6.1 Visual control condition

A first control analysis was performed using the *visual control* condition. To ensure that our results could not be explained by the rating procedure, we repeated both the whole electrode and the source space analysis performed for the *spontaneous pain* condition using data from the *visual control* condition. Here, ratings represented the continuously estimated length of the visually presented red bar instead of the currently perceived pain intensity. Based on these ratings, linear regressions with brain activity in theta, alpha, beta, and gamma frequency bands were again calculated for each electrode/voxel. The resulting coefficients were statistically tested for the *visual control* condition and contrasted with the corresponding coefficients from the *spontaneous pain* condition (see below). For a visualization of the frequency spectrum of relationships up to 100 Hz, the frequency‐resolved analysis at electrode Fz was also repeated for the *visual control* condition. Data from the *visual control* condition of one subject were not available due to technical difficulties during the recording. Thus, data from this subject were excluded from all analyses involving the *visual control* condition.

#### 2.6.2 Muscle activity

Further control analyses focused on possible confounds of gamma oscillations by muscle activity (Hipp & Siegel, 2013; Muthukumaraswamy, 2013). By applying a thorough artifact rejection using independent component analysis and beamformer‐based source localization, our analysis followed recent recommendations aiming to reduce potential confounding influences of muscle artifacts on estimates of high‐frequency brain activity (Hipp & Siegel, 2013; Muthukumaraswamy, 2013). Additionally, it has been suggested to simultaneously record activity from EMG electrodes and subject these to the same analysis as the main signal of interest to show that effects of interest are restricted to the signal of interest and not found for EMG data (Gross et al., 2013). Thus, we subjected data from both neck and masseter EMG electrodes to the same analysis as the EEG electrodes by computing linear regressions between the average activity in the gamma frequency band and bin orders based on the sorted pain ratings for the *spontaneous pain* condition and performing equivalent statistics (see below).

In addition, we performed an analysis of data rejected as artifact components after independent component analysis during preprocessing. As these artifact components likely include significant muscle activity, we were interested to know whether we would observe similar relationships between these data and pain intensity as found for artifact‐cleaned data. The same preprocessing pipeline as before was used but now, all independent components previously classified as clean were subtracted from the raw, unfiltered EEG data, retaining only data based on independent components classified as artifact contaminated. Source analysis of relationships between pain ratings in the *spontaneous pain* condition and activity in the gamma frequency band was then repeated and, as before, relationships between pain ratings and gamma amplitudes were quantified and statistically tested.

Last, we recomputed our electrode space analysis using a surface Laplacian referencing scheme instead of the previously used average referencing approach. Based on weighted referencing according to interelectrode distances, the surface Laplacian aims at attenuating low spatial frequency components in the data and improving topographical localization and has been suggested as a tool to reduce EMG contamination (Fitzgibbon et al., 2013; Fitzgibbon et al., 2015). After preprocessing and before further analysis, Laplacian rereferencing was performed using the spherical spline method (Perrin, Pernier, Bertrand, & Echallier, 1989) and analyses quantifying relationships between ratings and brain activity in the different frequency bands were repeated.

#### 2.6.3 Medication

To investigate a potential link between our observed effects and the patient's medication, we used the medication quantification score (MQS) of every patient (see Table 1), which summarizes pain‐related medications depending on their dosage and their potential to cause adverse effects (Harden et al., 2005). Using linear regressions, these scores were related to single subject beta values quantifying the strength of the relationship between ongoing pain and gamma power at electrode Fz in the *spontaneous pain* condition (see Table 2).

**Table 2:** Single‐subject data quantifying the relationship between ongoing pain intensity and gamma oscillations in the *spontaneous pain* condition at electrode Fz

Subject	Beta	SE	*p* value	*R* ^2^
1	.07	.09	.51	.16
2	−.02	.17	.92	.00
3	.23	.04	.01	.92
4	.19	.07	.07	.72
5	.02	.01	.15	.55
6	.12	.07	.19	.49
7	.21	.07	.05	.76
8	.00	.03	.89	.01
9	.08	.03	.08	.69
10	−.05	.05	.41	.23
11	−.02	.01	.25	.40
12	−.03	.07	.68	.07
13	.10	.07	.23	.43
4	.19	.06	.04	.79
15	.23	.03	0.00	.96
16	.14	.15	0.42	.23
17	−.09	.03	0.07	.71
18	.11	.07	0.18	.50
19	−.15	.06	0.08	.70
20	−.03	.04	0.48	.18
21	.39	.08	0.01	.90
22	.09	.06	0.24	.42
23	.24	.03	.00	.95
24	.04	.04	.45	.20
25	−.05	.05	.39	.25
26	−.04	.10	.72	.05
27	.30	.10	.05	.76
28	.04	.03	.25	.41
29	.12	.06	.13	.59
30	−.01	.05	.91	.00
31	.18	.13	.27	.38
Mean	.08	.06		

Beta = linear regression coefficient; R^2^ = explained variance; SE = standard error.

### 2.7 Statistical analysis

With the exception of the trend analysis (see below), the same general nonparametric (cluster‐based) permutation approach (Maris, 2012; Maris & Oostenveld, 2007) based on a dependent‐samples regression *t* statistic (Litvak et al., 2007; Lorch & Myers, 1990) was used with slight adaptations to statistically test relationships of ratings with frequency band specific activity for EEG and EMG data on electrode and source level. The applied cluster‐based procedure deals with the multiple comparison problem and is not affected by partial dependence in the data (Maris & Oostenveld, 2007). All statistical tests were two‐sided with a significance level of .05.

For the statistical analysis of the *spontaneous pain* and *visual control* condition on electrode level, dependent‐samples regression *t* statistics quantifying the relations between ratings and brain activity were computed as described above. Next, statistical significance was evaluated using cluster‐based permutation statistics. Clusters of neighboring electrodes, whose *t* statistic exceeded a critical threshold of *p* = .05, were selected and *t* values within each cluster were summed up, resulting in cluster‐level test statistics. The maximum cluster‐level test statistic was then compared to a reference distribution of maximum cluster *t* value sums obtained by randomly interchanging the bin labels and recalculating the cluster‐level test statistic 1,000 times. This comparison resulted in a *p* value per condition and frequency band, which was given by the proportion of permutations in which the maximum cluster‐level test statistic exceeded the actually observed maximum cluster‐level test statistic in the data. For the analysis of relations between ratings and brain activity in the different frequency bands on source level, the same procedure was used, but clusters were formed across voxels instead of electrodes.

In addition, to directly compare the relationships of ratings with brain activity between the *spontaneous pain* and *visual control* conditions on both electrode and source level, the single subject regression coefficients obtained from both conditions were contrasted for each frequency band by computing dependent‐samples *t* tests comparing the individual regression coefficients between the two conditions. Cluster‐level test statistics were again calculated based on the sum of *t* values in clusters of neighboring electrodes/voxels, whose *t*‐statistic exceeded a critical threshold of *p* = .05. For each frequency band, the reference distribution for the maximum cluster‐level test statistic was here obtained by swapping the single subject regression coefficients from the *spontaneous pain* and *visual control* condition for a random subset of *n* = 30 subjects and recalculating the cluster‐level test statistic 1,000 times instead of randomly interchanging bin labels.

Last, the same statistical approach was used for the control analyses of muscle activity. For the two EMG electrodes, the same permutation analysis as for the single condition electrode level was used, comparing the original dependent‐samples regression *t* statistics with a distribution of dependent‐samples regression *t* values obtained after randomly permuting the bin labels 1,000 times. As only a single electrode was investigated at a time, the test statistic was now based on the single electrode *t* statistic instead of cluster *t*‐value sums. For a comparison of the obtained *t* and *p* values of both neck and masseter electrodes with those of an exemplary single EEG electrode, the same analysis was also performed for the fronto‐central EEG electrode Fz. Finally, relations between ratings and activity in the gamma frequency band for the part of the data of the *spontaneous pain* condition previously rejected as artifact contaminated were statistically tested using the same source level statistical approach described for the analysis of the cleaned data.

Finally, the type of relationship between ongoing pain and gamma activity in the *spontaneous pain* condition was analyzed at electrode Fz using repeated measures ANOVA with subsequent standard trend analysis as implemented in SPSS. The five pain rating bins were used as within‐subject factor for the repeated measures ANOVA, which was followed by tests for linear, quadratic, cubic, and quartic trends.

## 3 RESULTS

### 3.1 Behavioral data

Figure 1 shows the time courses of chronic back pain intensity ratings in the *spontaneous pain* condition for all participants. In line with previous studies ((Baliki et al., 2006; Baliki et al., 2011; Foss, Apkarian, & Chialvo, 2006), behavioral data showed spontaneous fluctuations of ongoing pain over the course of the experiment. Because the strength of these fluctuations varied between patients, ratings were z‐transformed for each subject. Based on the visual analogue scale anchored at *no pain* (0) and *worst imaginable pain* (100) and the original units, mean pain intensity averaged across the analyzed time window and then across participants was 41 ± 21 (mean ± standard deviation). Pain ratings significantly increased over the course of the experiment (mean (± standard deviation) pain ratings first half: 39 (± 20), second half: 43 (± 24); dependent‐samples *t* test: *T*_(30)_ = 2.80, *p* = .009). The mean current pain intensity rated immediately before the experiment was 52 ± 16, the mean chronic pain duration 11 ± 9 years. Please see Table 1 for detailed patient characteristics and results from questionnaires.

### 3.2 Neurophysiological representation of ongoing back pain intensity

**Figure 2:**
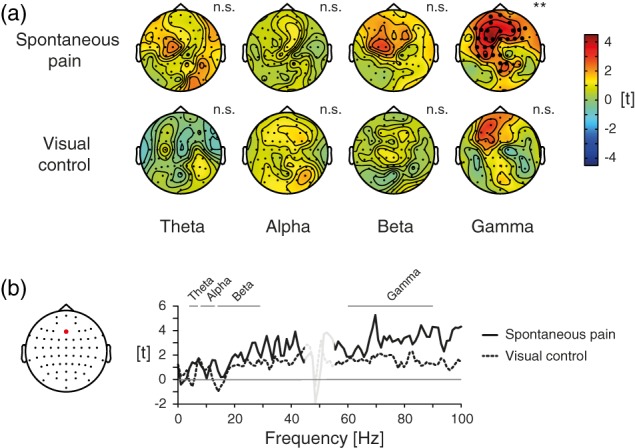
Relationships between ongoing pain intensity and neuronal oscillations on electrode level. (a) Electrode level *t* maps of the relationship between ratings during the *spontaneous pain* and *visual control* condition and brain activity as assessed by linear regressions for theta (4–7 Hz), alpha (8–13 Hz), beta (14–29 Hz), and gamma (60–90 Hz) frequencies. Scaling reflects *t* values resulting from nonparametric cluster‐based permutation tests. Positive and negative relationships are reflected by warm and cold colors, respectively. Electrodes within significant clusters are marked. n.s., not significant; **p < .01 (two‐sided). (b) The right panel descriptively displays the frequency spectrum of the relationship between pain intensity and brain activity in the *spontaneous pain* and *visual control* condition for electrode Fz, which is highlighted in the topography in the left panel. Again, *t* values are shown. Frequency bands used in all analyses are marked. The strongest (positive) relationship was observed at 70 Hz. Relationships between 45 and 55 Hz, which are contaminated by line noise artifacts, are masked. [Color figure can be viewed at http://wileyonlinelibrary.com]

**Figure 3:**
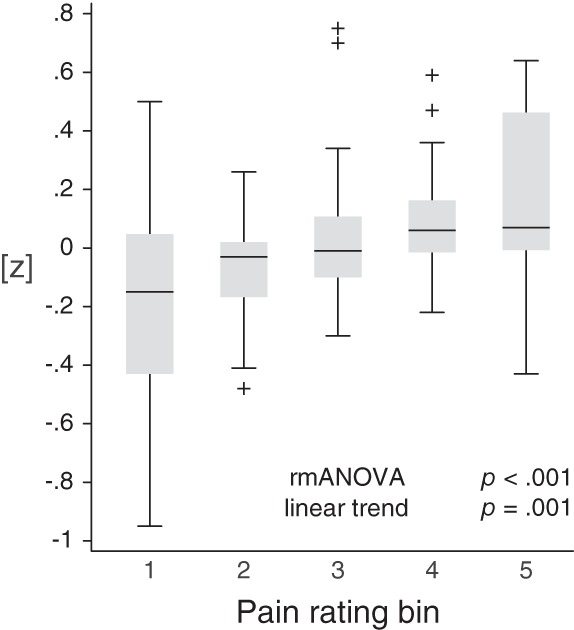
Trend analysis of the relationship between ongoing pain intensity and neuronal oscillations in the gamma frequency band. Box plots of individual, *z*‐transformed gamma activity at electrode Fz in the *spontaneous pain* condition are shown, sorted into five bins based on pain ratings. Gamma activity increased with increasing pain. A repeated measures ANOVA revealed a significant main effect of the bin number (*F*_(3, 80)_ = 7.23, *p* < .001). A subsequent trend analysis showed a significant linear trend (*F*_(1, 30)_ = 13.62, *p* = .001), while quadratic, cubic, and quartic trends were not significant (quadratic: *F*_(1, 30)_ = 1.13, *p* = .30; cubic: *F*_(1, 30)_ = .11, *p* = .74; quartic: *F*_(1, 30)_ = .48, *p* = .49). rmANOVA, repeated measures analysis of variance.

We first investigated how neuronal activity in different frequency bands reflects ongoing pain intensity on electrode level. Please see Supporting Information, Figure S1 for topographies of raw amplitudes in the different frequency bands and raw power spectra of brain activity at electrode Fz. Using five data bins sorted by pain intensity, we calculated linear regressions quantifying the relationships between the continuously rated current back pain intensity and the amplitude of brain activity in each frequency band for each electrode in the *spontaneous pain* condition. Regression coefficients were statistically tested using cluster based permutation statistics, resulting in electrode level *t* value maps for each frequency band, which are shown in Figure 2a (upper row). The analysis revealed a significant cluster of positive relationships between pain ratings and gamma power at frontal electrodes (*p* = .005, marked by black circles). Thus, higher pain ratings were associated with stronger frontal gamma oscillations. Table 2 shows the single‐subject regression coefficients, standard errors, *p* values, and estimates of the explained variance for the frontal electrode Fz. No significant relationships were observed between pain ratings and brain activity in theta, alpha, or beta frequency bands (*p* > .05 for all clusters, two‐sided). An analysis without previous *z*‐transformation of ratings and EEG data showed the same pattern, confirming a significant frontal gamma effect in the *spontaneous pain* condition only (data not shown). A frequency resolved analysis of the relationship between brain activity and ongoing pain intensity confirmed that the strongest relations were found above 30 Hz in the gamma frequency range (Figure 2b) with a peak at 70 Hz. Further analyses up to 200 Hz indicated that differences between the conditions continued in higher frequencies but strongest relationships were found below 100 Hz (data not shown). As can be seen in Figure 3, a trend analysis at electrode Fz confirmed a significant linear relationship between ongoing pain and gamma activity (repeated measures ANOVA: *F*_[3, 80]_ = 7.23, *p* < .001; linear trend: *F*_[1, 30]_ = 13.62, *p* = .001) but did not show evidence for a quadratic, cubic or quartic relation (quadratic: *F*_(1, 30)_ = 1.13, *p* = .30; cubic: *F*_(1, 30)_ = .11, *p* = .74; quartic: *F*_(1, 30)_ = .48, *p* = .49).

Two further analyses revealed a contribution of time to the observed relationship between gamma activity and pain (Supporting Information, Figure S2). A split half analysis of the first and second 4.5 min of the analyzed time window revealed a significant cluster of positive relationships between pain ratings and gamma power at frontal electrodes for the first half of the *spontaneous pain* condition only but not for the second half or the *visual control* condition. Quantifying relationships between ratings and gamma power controlling for time, no significant clusters of relationships between ratings and gamma power were found. Together with the significant pain rating increase across the experiment, these analyses suggest that the positive relationship between pain ratings and frontal gamma power was coupled to a slow increase of pain ratings and gamma power over the course of the recording session (Figure 1).

**Figure 4:**
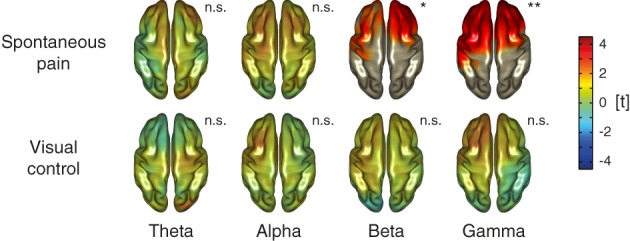
Relationships between ongoing pain intensity and neuronal oscillations on source level. Source‐level *t* maps of the relationship between ratings during the *spontaneous pain* and *visual control* condition and brain activity as assessed by linear regressions for theta (4–7 Hz), alpha (8–13 Hz), beta (14–29 Hz), and gamma (60–90 Hz) frequencies. As in the previous figure, scaling reflects *t* values resulting from nonparametric cluster‐based permutation tests and positive and negative relationships are reflected by warm and cold colors, respectively. In plots showing significant relationships, areas outside of significant clusters are masked. in plots without significant effects, opacity is reduced. n.s., not significant; *p < .05 (two‐sided), **p < .01 (two‐sided). [Color figure can be viewed at http://wileyonlinelibrary.com]

In the next step, we determined where the significant relationships between pain ratings and gamma oscillations were localized in the brain. Using LCMV‐based source analysis, time courses of frequency band specific activity in the *spontaneous pain* condition were projected from electrode to source level and linear regressions and statistical analyses were repeated on voxel level (Figure 4, upper row). This analyses revealed significant clusters of positive relationships between ongoing pain and beta (*p* = .024) as well as gamma oscillations (*p* = .008). Both clusters had a similar shape covering bilateral frontal and prefrontal brain areas. Please see Supporting Information, Figure S3 for additional views of the relationships in the gamma band. No significant relationships were observed for brain activity in theta and alpha frequency bands.

### 3.3 Control analyses

To control for activity related to the continuous rating procedure such as visual‐motor performance, magnitude estimation, and anticipation (Baliki et al., 2011; Hashmi et al., 2013; Nickel et al., 2017), we performed a *visual control* condition asking participants to continuously rate the length of a visual bar instead of the ongoing pain intensity. Unknown to the subject, the bar length replayed the time course of the individual pain rating from the *spontaneous pain* condition. Using this rating and corresponding EEG data, both electrode and source level analyses of the relationships between rating and brain activity were repeated. In contrast to the results from the *spontaneous pain* condition, no significant relationships were observed for theta, alpha, beta, or gamma frequency bands on electrode (Figure 2a, lower row) or source level (Figure 4, lower row) in the *visual control* condition (all *p* > .05, two‐sided). The direct statistical contrast of regression coefficients from both conditions on electrode or source level did not reveal significant differences in any frequency band (all *p* > .05, two‐sided).

**Figure 5:**
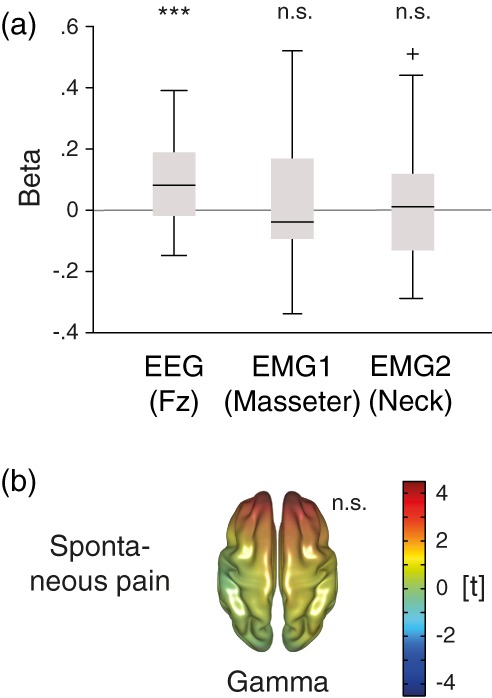
Control analyses of muscle activity. (a) Relationships between pain ratings and activity in the gamma frequency band (60–90 Hz) in the *spontaneous pain* condition are shown for the exemplary fronto‐central EEG electrode Fz (see left panel in Figure 2b) and two EMG electrodes placed on the right masseter and neck muscles. As in previous figures, *t* values resulting from nonparametric cluster‐based permutation tests based on linear regressions are shown. EEG, electroencephalography, EMG, electromyography, n.s., not significant; ***p < .001. (b) Source‐level *t* map of the relationship between ratings during the *spontaneous pain* condition and data reconstructed from independent components, which were classified as artifact‐contaminated during preprocessing. Relationships based on linear regressions are shown for the gamma frequency band (60–90 Hz). As in previous figures, scaling reflects *t* values resulting from nonparametric cluster‐based permutation tests, positive and negative relationships are reflected by warm and cold colors, respectively, and opacity is reduced as no significant effects were found. n.s., not significant. [Color figure can be viewed at http://wileyonlinelibrary.com]

To control for possible confounds by muscle activity, we conducted three additional analyses. First, analyses of the relationship between pain ratings and gamma activity at EMG electrodes attached to the neck and masseters muscles were performed. No significant associations were found (*p* > .05, two‐sided, Figure 5a). Second, source level relationships between activity in the gamma frequency band and pain ratings were repeated for that part of the data previously rejected as artifacts. No significant relationships between gamma amplitudes and pain ratings were found (p > .05, two‐sided, Figure 5b). Third, electrode space analyses were repeated based on a surface Laplacian referencing scheme (Supporting Information, Figure S4). In addition to a significant cluster of positive relationships of ratings in the *spontaneous pain* condition to frontal gamma power (*p* = .013, two‐sided), this approach also revealed a cluster of positive relationships to frontal beta power (*p* < .001, two‐sided). Thus, potentially better controlling for EMG contamination than the original average referencing approach (Fitzgibbon et al., 2013; Fitzgibbon et al., 2015), this analysis confirmed our previous findings.

Last, we investigated a potential link between the strength of relationship between pain ratings and gamma power at Fz and the patients' medication measured by the medication quantification scores (MQS). Linear regressions did not show a significant relation between the two (*ß* = 0.002, *p* = .45). This analysis did therefore not provide evidence for a confounding effect of medication on the observed relationships.

## 4 DISCUSSION

This study investigated the neurophysiological representation of ongoing pain in chronic back pain patients. Continuous pain ratings confirmed spontaneous fluctuations of ongoing pain already within minutes. EEG data revealed a positive association between ongoing pain intensity and the amplitude of prefrontal beta and gamma oscillations, which have been related to the perception of longer lasting experimental pain (Nickel et al., 2017; Schulz et al., 2015). In contrast, no significant relationship between neuronal alpha and beta oscillations in sensorimotor areas were found, which have been related to the processing of nociceptive information (Nickel et al., 2017; Schulz et al., 2015). These findings provide physiological support for a dissociation of ongoing pain from nociceptive processes in chronic pain (Baliki & Apkarian, 2015). Moreover, they hint at prefrontal gamma oscillations as a potential neurophysiological marker of ongoing pain as the key symptom of chronic pain.

A role of prefrontal areas in the encoding of ongoing pain is in line with results from previous fMRI studies investigating different chronic pain populations (Baliki et al., 2006; Baliki et al., 2011; Geha et al., 2007; Hashmi et al., 2013; Parks et al., 2011). Furthermore, fronto‐striatal circuits and the prefrontal cortex have been implicated in the estimation of subjective value (Clithero & Rangel, 2014; Grabenhorst & Rolls, 2011) and affective meaning (Roy, Shohamy, & Wager, 2012) across different stimuli, tasks, and modalities. Moreover, changes of these circuits are often observed in neuropsychiatric disorders, which are associated with a negative emotional state (Kaiser, Andrews‐Hanna, Wager, & Pizzagalli, 2015; Russo & Nestler, 2013). Psychiatric disorders such as depression and chronic pain frequently co‐occur (Velly & Mohit, 2018) and 19 out of 31 of our participants also showed depression scores in line with at least mild to moderate depression (Table 1). Altogether, a role of prefrontal areas in the encoding of ongoing pain fits well with previous findings and points to an important function of emotional‐evaluative rather than primary sensory neural circuits in chronic pain.

The relationship of ongoing pain intensity to neuronal oscillations at gamma frequencies corresponds to results from previous studies, which showed a representation of the intensity of ongoing experimental pain by prefrontal gamma oscillations in healthy participants (Nickel et al., 2017; Schulz et al., 2015). Gamma band oscillations are found in many different brain areas and have been associated with a broad range of cognitive and behavioral functions including object representation, memory, and attention (Donner & Siegel, 2011; Fries, 2015; Wang, 2010). Thus, they likely represent a basic feature of neuronal signaling and communication (Donner & Siegel, 2011; Fries, 2015; Wang, 2010). Gamma oscillations appear to be particularly related to feedforward communication and the transmission of currently important stimuli (Donner & Siegel, 2011; Fries, 2015; Ploner, Sorg, & Gross, 2017). These concepts would be in line with a positive association of ongoing pain intensity with gamma oscillations. We also found a positive relationship of ongoing pain intensity and frontal beta oscillations. However, the topography of this relationship was similar to that between pain intensity and gamma oscillations. Moreover, the frequency spectrum of the relationships between pain intensity and brain activity shows strongest effects at gamma frequencies. It is therefore likely that the relationships between pain intensity and neuronal oscillations at gamma and beta oscillations essentially represent similar underlying mechanisms.

The potential use of oscillations as biomarkers in clinical studies has previously been discussed (Basar & Guntekin, 2013) and oscillations represent a promising target for clinical interventions such as neurofeedback and neurostimulation (Jensen et al., 2014; Sitaram et al., 2017; Thut et al., 2017). In comparison to markers reflecting persistent changes of brain function in chronic pain, a neurophysiological marker reflecting the dynamics of ongoing pain intensity would be of particular interest in this respect. However, due to their small amplitude and potential confounding artifacts in noninvasive recordings, the signal‐to‐noise ratio of high‐frequency oscillations is comparably low, challenging the precise quantification on the individual subject level. Future studies will thus need to show whether prefrontal gamma oscillations can be used as a diagnostic marker of ongoing pain intensity and whether the targeted reduction of gamma oscillations can be used to reduce ongoing pain.

In previous studies investigating ongoing experimental pain in healthy humans, we found a significant relationship between objective stimulus intensity and neuronal oscillations over primary sensorimotor areas (Nickel et al., 2017; Schulz et al., 2015). In this study, we did not observe similar relationships suggesting that ongoing pain intensity can dissociate from nociceptive processes in chronic pain patients. However, this does not preclude a relevance of sensory information for ongoing pain in chronic pain, which might not be detectable using the current EEG approach.

Muscle activity represents an important confound of high frequency activity recorded by EEG (Hipp & Siegel, 2013; Muthukumaraswamy, 2013). Separation of brain activity from muscle activity is particularly challenging as the topography, frequency, and amplitude of muscle artifacts differ across participants, muscles, and the direction and force of contraction (Goncharova, McFarland, Vaughan, & Wolpaw, 2003; Kumar, Narayan, & Amell, 2003; O'Donnell, Berkhout, & Adey, 1974; Yuval‐Greenberg, Tomer, Keren, Nelken, & Deouell, 2008). Thus, it is not possible to define a single, clear criterion for disentangling brain activity from muscle activity. Instead, the separation of brain activity and muscle activity can only depend on a mosaic of evidence from careful artifact rejection procedures (e.g., ICA‐based), analysis strategies (e.g., source space, Laplacian), and control analyses (e.g., analysis of EMG electrodes) (Gross et al., 2013; Muthukumaraswamy, 2013). We performed artifact correction according to recent guidelines (Hipp & Siegel, 2013; Muthukumaraswamy, 2013), did not find significant relationships between gamma amplitudes of two EMG electrodes as well as artifact‐dominated data and ongoing pain (Gross et al., 2013), and confirmed our findings using a surface Laplacian referencing scheme. However, no method can guarantee data free of high‐frequency artifacts (Muthukumaraswamy, 2013) and even additional EMG electrodes closer to the forehead would likely pick up activity from both muscle and brain. Thus, we cannot ultimately rule out muscle confounds in this study.

Some further limitations have to be considered in relation to the interpretation of the present findings. First, significant relationships between neuronal oscillations and ratings were found for the *spontaneous pain* but not for the *visual control* condition. However, the direct contrast of the two conditions was not significant. A potential explanation could be the slow increase of pain levels in the *spontaneous pain* condition over the course of the experiment. In the *visual control* condition, the pain ratings from the *spontaneous pain* condition were replayed. Assuming that some patients again experienced a slow increase of pain during the *visual control* condition, that is, while they were seated and could not move freely, pain and ratings in the *visual control* condition might have again co‐varied so that part of the effects in the *visual control* condition might eventually reflect relationships between gamma oscillations and ongoing pain. This would not preclude true gamma‐pain relations but limit the power of the condition contrast. Second, further analyses indicated a significant contribution of time and slow increases of pain during the recordings to the observed relationships between pain ratings and frontal gamma power (Supporting Information, Figure S2). Considering that patients were asked to sit as still as possible, this steady increase of pain and the resulting time confound seems plausible. Again, this does not argue against true gamma‐pain relationships but indicates that the slow increase rather than faster pain fluctuations largely carried the effect. Third, brain activity might always also encode other aspects, which co‐vary with perceived pain such as unpleasantness, salience, or changes in the attentional state. Albeit the standard in previous similar studies (Baliki et al., 2011; Hashmi et al., 2013; Nickel et al., 2017), the task in the *visual control* condition clearly differed from the *spontaneous pain* condition asking patients to rate a visual stimulus rather than an internal state. Developing a control condition in which patients monitor another ongoing, ideally equally salient and also internal sensation would be highly desirable. Moreover, our results do not necessarily generalize to other recording conditions, for example, with eyes closed, and we cannot completely rule out effects due to the fixed order of our two conditions. For example, task difficulty might have decreased over time, resulting in a higher working memory load earlier on in the experiment. Fourth, the relation between gamma oscillations and pain intensity was not focal but wide‐spread, especially in source space. Thus, no strong claims about the exact location are possible. However, the analyses localized the relation to prefrontal areas and thus beyond primary sensorimotor areas, which are implicated in the encoding of phasic pain and nociceptive stimulus input (Nickel et al., 2017; Schulz et al., 2015). Last, further studies need to investigate if ongoing pain in other chronic pain conditions is also reflected by prefrontal gamma oscillations. Previous fMRI studies have shown an involvement of comparable brain regions in the representation of spontaneous pain across distinct pain populations (Baliki et al., 2011; Geha et al., 2007; Hashmi et al., 2013; Parks et al., 2011), making similar underlying mechanisms plausible.

## 5 CONCLUSIONS

Taken together, the current results indicate that prefrontal gamma oscillations reflect the intensity of ongoing pain in chronic back pain patients. Thus, they reveal a potential neurophysiological marker of ongoing pain, which could be measured relatively easily using EEG as a noninvasive and broadly available clinical tool. They are furthermore in line with a role of emotional‐evaluative circuits rather than sensory circuits in ongoing pain, emphasizing the emotional aspects of the chronic pain experience. Future studies need to take potential muscle confounds into account, but might investigate the potential of prefrontal gamma activity as a marker of ongoing pain for the diagnosis and treatment of chronic pain (Davis et al., 2017), especially in the context of neurofeedback and neurostimulation treatment approaches (Jensen et al., 2014; Sitaram et al., 2017; Thut et al., 2017).

## Supporting information


**Figure S1** Frequency specific topographies and power spectra of EEG signals. (A) Topographies of amplitudes of brain activity for theta (4–7 Hz), alpha (8–13 Hz), beta (14–29 Hz) and gamma (60–90 Hz) frequencies. Amplitudes were averaged across the whole analyzed time window and all subjects for the *spontaneous pain* and the *visual control* condition separately. (B) The right panel shows power spectra of brain activity in the *spontaneous pain* and the *visual control* condition at electrode Fz, which is highlighted in the topography in the left panel. Based on 1 s segments with 90% overlap, power was estimated for frequencies between 1 and 100 Hz in steps of 1 Hz using the Fast Fourier Transform after applying a Hanning taper. Grand averages across all segments of the analyzed time window and all subjects are shown. Frequency bands used in all analyses are marked. Please note that the peak around 50 Hz reflects line noise.
**Figure S2**. Contribution of time to the observed relationship between ratings and gamma power. Electrode level t‐maps of the relationship between ratings during the *spontaneous pain* and *visual control* condition and gamma power (60 to90 Hz) as assessed by linear regressions. The first column shows results from the original analysis (see Figure 4(a) of the manuscript). The second and third column show results for the first and second half of the data, respectively. The fourth column shows relationships when controlling for time since the start of recording within the regression models. Scaling reflects t‐values resulting from nonparametric cluster‐based permutation tests. Positive and negative relationships are reflected by warm and cold colors, respectively. Electrodes within significant clusters are marked. n.s., not significant; *p < .05 (two‐sided), **p < .01 (two‐sided).
**Figure S3**. Relationship between ongoing pain intensity and neuronal activity in the gamma frequency band in the spontaneous pain condition on source level. Additional views of the observed significant cluster of relationships between pain ratings and gamma power (60 to 90 Hz) on source level are shown (see Figure 3). Scaling reflects t‐values resulting from nonparametric cluster‐based permutation tests, positive and negative relationships are reflected by warm and cold colors, respectively. Areas outside of the significant cluster (p < .01, two‐sided) are masked.
**Figure S4**. Relationships between ongoing pain intensity and neuronal oscillations on electrode level using a Laplacian reference scheme. Electrode level t‐maps of the relationship between ratings during the *spontaneous pain* and *visual control* condition and brain activity as assessed by linear regressions for theta (4–7 Hz), alpha (8–13 Hz), beta (14–29 Hz) and gamma (60–90 Hz) frequencies. In contrast to the previously used average reference approach, data analysis was here based on a Laplacian reference scheme. Scaling reflects t‐values resulting from nonparametric cluster‐based permutation tests. Positive and negative relationships are reflected by warm and cold colors, respectively. Electrodes within significant clusters are marked. n.s., not significant; *p < .05 (two‐sided), **p < .01 (two‐sided).
