# Supplementary material for: Prefrontal gamma oscillations reflect ongoing pain intensity in chronic back pain patients
Source: Hum Brain Mapp. 2018 Sep 10;40(1):293–305. doi: 10.1002/hbm.24373 (PMC6585682; doi:10.1002/hbm.24373)
Supplement: Supplementary file 1 — Figure S1 Frequency specific topographies and power spectra of EEG signals. (A) Topographies of amplitudes of brain activity for theta (4–7 Hz), alpha (8–13 Hz), beta (14–29 Hz) and gamma (60–90 Hz) frequencies. Amplitudes were averaged across the whole analyzed time window and all subjects for the spontaneous pain and the visual control condition separately. (B) The right panel shows power spectra of brain activity in the spontaneous pain and the visual control condition at electrode Fz, which is highlighted in the topography in the left panel. Based on 1 s segments with 90% overlap, power was estimated for frequencies between 1 and 100 Hz in steps of 1 Hz using the Fast Fourier Transform after applying a Hanning taper. Grand averages across all segments of the analyzed time window and all subjects are shown. Frequency bands used in all analyses are marked. Please note that the peak around 50 Hz reflects line noise. Figure S2. Contribution of time to the observed relationship between ratings and gamma power. Electrode level t‐maps of the relationship between ratings during the spontaneous pain and visual control condition and gamma power (60 to90 Hz) as assessed by linear regressions. The first column shows results from the original analysis (see Figure 4(a) of the manuscript). The second and third column show results for the first and second half of the data, respectively. The fourth column shows relationships when controlling for time since the start of recording within the regression models. Scaling reflects t‐values resulting from nonparametric cluster‐based permutation tests. Positive and negative relationships are reflected by warm and cold colors, respectively. Electrodes within significant clusters are marked. n.s., not significant; *p < .05 (two‐sided), **p < .01 (two‐sided). Figure S3. Relationship between ongoing pain intensity and neuronal activity in the gamma frequency band in the spontaneous pain condition on source level. Additional views of the obs [file HBM-40-293-s001.docx]

**Supplementary Material for**

**Prefrontal gamma oscillations reflect ongoing pain intensity in chronic back pain patients**

Elisabeth S. May^1,2^, Moritz M. Nickel^1,2^, Son Ta Dinh^1,2^, Laura Tiemann^1,2^, Henrik Heitmann^1,2^, Isabel Voth^1^, Thomas R. Tölle^1^, Joachim Gross^3,4^, Markus Ploner^1,2^

^1^Department of Neurology, Technische Universität München, 81675 Munich, Germany

^2^TUM-Neuroimaging Center, Technische Universität München, 81675 Munich, Germany

^3^Institute of Neuroscience and Psychology, University of Glasgow, Glasgow, G12 8QQ, United Kingdom

^4^Institute for Biomagnetism and Biosignalanalysis, University of Münster, 48149 Münster, Germany


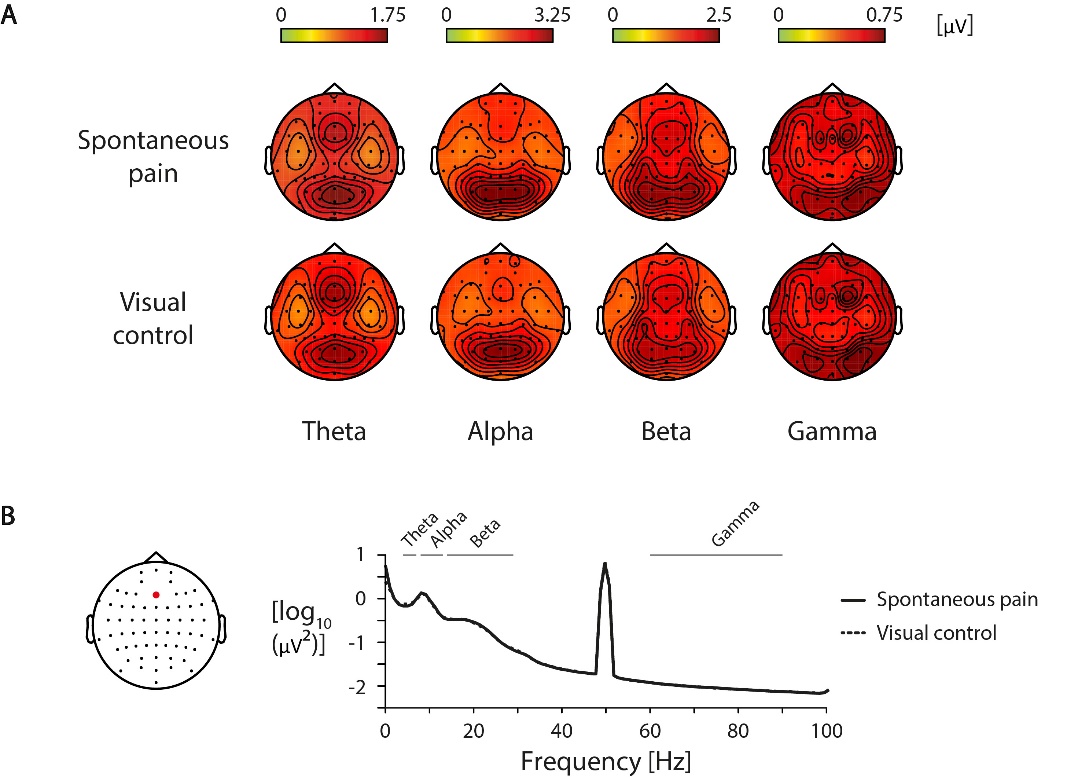


**Figure S1. Frequency specific topographies and power spectra of EEG signals. (A)** Topographies of amplitudes of brain activity for theta (4-7 Hz), alpha (8-13 Hz), beta (14-29 Hz) and gamma (60-90 Hz) frequencies. Amplitudes were averaged across the whole analyzed time window and all subjects for the spontaneous pain and the visual control condition separately. **(B)** The right panel shows power spectra of brain activity in the spontaneous pain and the visual control condition at electrode Fz, which is highlighted in the topography in the left panel. Based on 1 s segments with 90% overlap, power was estimated for frequencies between 1 and 100 Hz in steps of 1 Hz using the Fast Fourier Transform after applying a Hanning taper. Grand averages across all segments of the analyzed time window and all subjects are shown. Frequency bands used in all analyses are marked. Please note that the peak around 50 Hz reflects line noise.


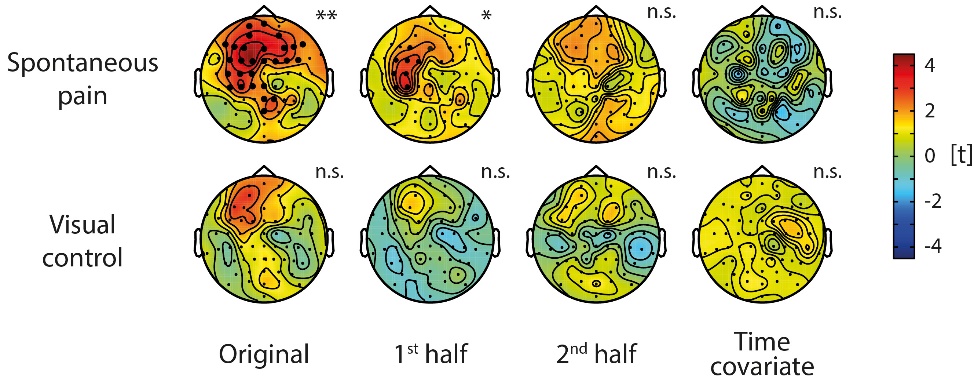


**Figure S2. Contribution of time to the observed relationship between ratings and gamma power.** Electrode level t-maps of the relationship between ratings during the spontaneous pain and visual control condition and gamma power (60 to90 Hz) as assessed by linear regressions. The first column shows results from the original analysis (see Figure 4A of the manuscript). The second and third column show results for the first and second half of the data, respectively. The fourth column shows relationships when controlling for time since the start of recording within the regression models. Scaling reflects t-values resulting from nonparametric cluster-based permutation tests. Positive and negative relationships are reflected by warm and cold colors, respectively. Electrodes within significant clusters are marked. n.s., not significant; *p < 0.05 (two-sided), **p < 0.01 (two-sided).


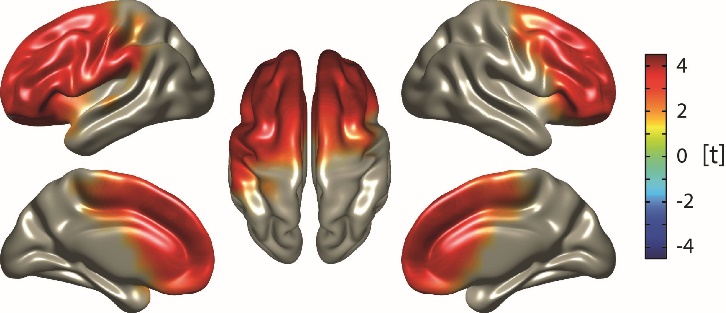


**Figure S3. Relationship between ongoing pain intensity and neuronal activity in the gamma frequency band in the spontaneous pain condition on source level.** Additional views of the observed significant cluster of relationships between pain ratings and gamma power (60 to 90 Hz) on source level are shown (see Figure 3). Scaling reflects t-values resulting from nonparametric cluster-based permutation tests, positive and negative relationships are reflected by warm and cold colors, respectively. Areas outside of the significant cluster (p < 0.01, two-sided) are masked.


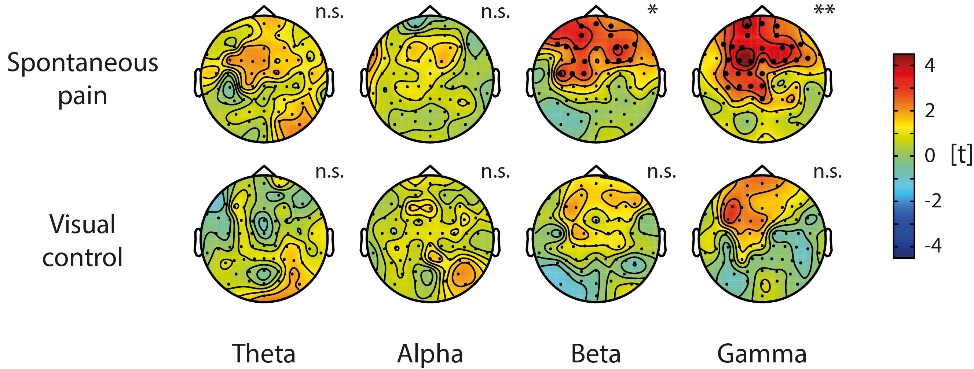


**Figure S4. Relationships between ongoing pain intensity and neuronal oscillations on electrode level using a Laplacian reference scheme.** Electrode level t-maps of the relationship between ratings during the spontaneous pain and visual control condition and brain activity as assessed by linear regressions for theta (4-7 Hz), alpha (8-13 Hz), beta (14-29 Hz) and gamma (60-90 Hz) frequencies. In contrast to the previously used average reference approach, data analysis was here based on a Laplacian reference scheme. Scaling reflects t-values resulting from nonparametric cluster-based permutation tests. Positive and negative relationships are reflected by warm and cold colors, respectively. Electrodes within significant clusters are marked. n.s., not significant; *p < 0.05 (two-sided), **p < 0.01 (two-sided).
